# Phytochemicals as Potential Therapeutics for SARS-CoV-2–Induced Cardiovascular Complications: Thrombosis and Platelet Perspective

**DOI:** 10.3389/fphar.2021.658273

**Published:** 2021-04-26

**Authors:** Samir K. Beura, Abhishek R. Panigrahi, Pooja Yadav, Sunil K. Singh

**Affiliations:** Department of Zoology, School of Basic and Applied Sciences, Central University of Punjab, Bathinda, India

**Keywords:** SARS-CoV-2, ACE2, thrombosis, platelet, myocardial injury, cytokine storm, phytochemical

## Abstract

After gaining entry through ACE2 aided by TMPRSS2, the SARS-CoV-2 causes serious complications of the cardiovascular system leading to myocarditis and other myocardial injuries apart from causing lung, kidney and brain dysfunctions. Here in this review, we are going to divulge the cellular and immunological mechanisms behind the cardiovascular, thrombotic and platelet impairments that are caused in COVID-19. In addition, we also propose the significance of various anti-platelet and anti-thrombotic phytochemicals in the treatment of COVID-19. The virus induces many immune-modulatory cytokines and chemokines which help in the intravascular coagulation and create a pro-thrombotic environment along with pulmonary embolism and thrombocytopenia. Different types of innate and adaptive immune cells and their granular contents regulate the pathophysiology of SARS-CoV-2 induced endothelial and platelet dysfunctions which correlate the involvement of platelets with myocardial injury and intravascular thrombi directly or indirectly. Hence, by exploiting the natural bioactive compounds from medicinal plants and inhibiting the platelet mediated thrombus formation can be beneficial for the treatment of SARS-CoV-2 infection.

## Introduction

**FIGURE 1:**
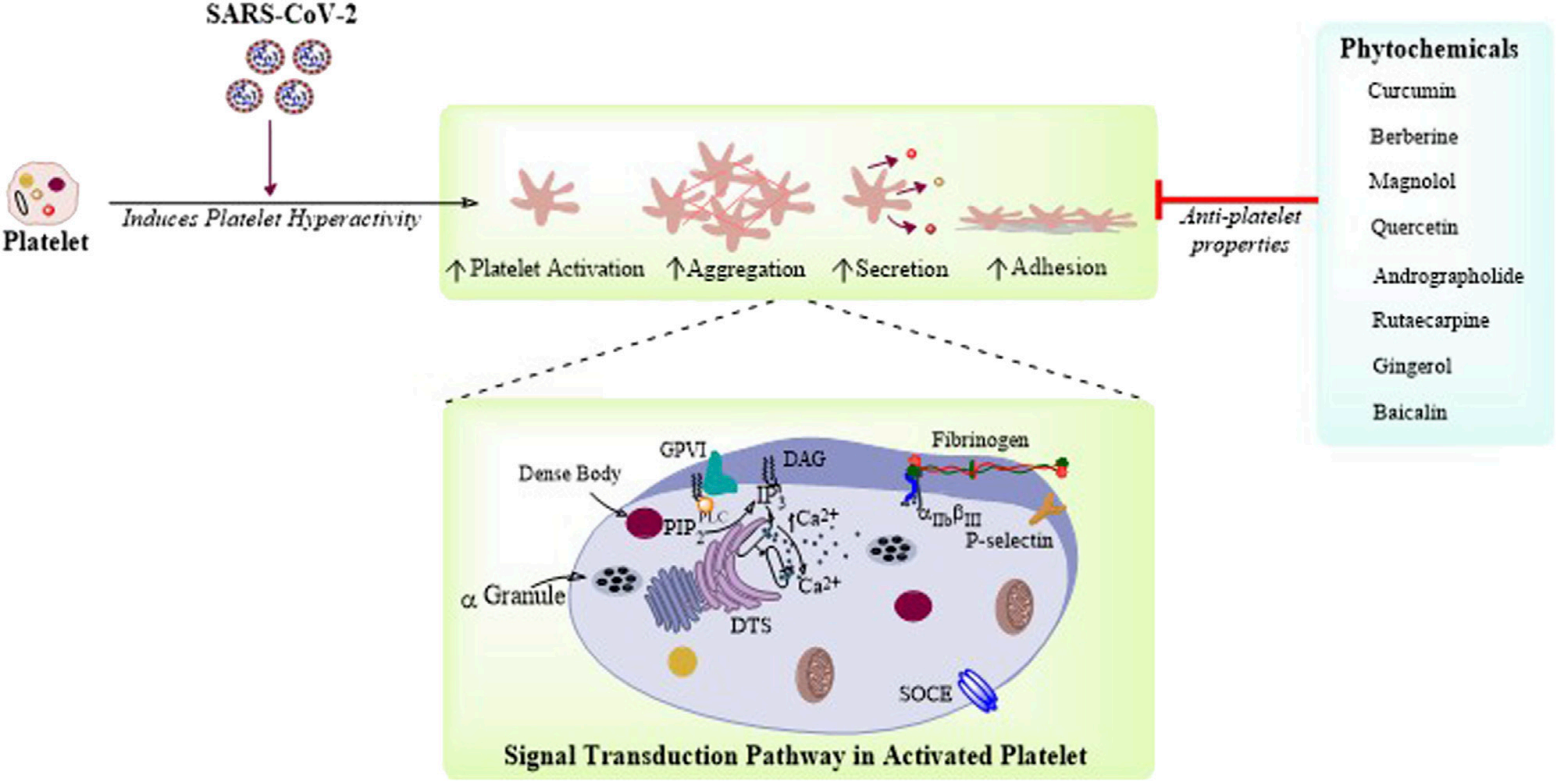
Phytochemicals targeting platelet function and thrombosis: Platelet function is governed by intracellular Ca^2+^ signaling resulting in platelet adhesion, activation, aggregation, and secretion. SARS-CoV-2 is demonstrated here to cause increased platelet activation, secretion, and aggregation. There are few phytochemicals which are reported to have antiplatelet activity and could be potential therapeutic candidates for the treatment of COVID-19.

The coronavirus disease 2019 (COVID-19) is a severe acute respiratory syndrome coronavirus 2 (SARS-CoV-2) caused disease that has reported a total of 119,222,995 infections and 2,644,461 deaths worldwide (as per the WHO report till March 15, 2021). The SARS-CoV-2 belongs to the genus Betacoronavirus, which is closely related to other recent past viruses of this group like severe acute respiratory syndrome coronavirus (SARS-CoV) and Middle East respiratory syndrome coronavirus (MERS-CoV) (Gao et al., 2020). The SARS-CoV-2 gains entry into the host cell primarily by the interaction of viral spike (S) glycoprotein with a host cell receptor that is, angiotensin converting enzyme 2 (ACE2), aided by the priming of S protein by a serine protease that is, transmembrane protease serine 2 (TMPRSS2) (Hoffmann et al., 2020b). Due to the abundance of ACE2 receptors in major tissues like pulmonary tissues, cardiac tissues, and associated endothelial tissues, there are more chances of developing viral-induced complications such as myocardial injuries and acute coronary injuries (Clerkin et al., 2020). The elevated concentration of immunomodulatory molecules like interleukins (IL-2 and IL-7), interferon (IFN-γ), G-CSF, MCP-1, MIP-1α, and tumor necrosis factor (TNF-α) (Table 1) in plasma is found in COVID-19 patients suggesting the cytokine storm–induced inflammation causing cardiac as well as pulmonary injuries (Mehta et al., 2020). This cytokine storm augments the expression of adhesion molecules which not only activates the endothelial cells inducing vascular inflammation but also causes inflammatory cell infiltration (e.g., macrophages). The dysregulation of cytokines causes vascular endothelial dysfunction, thus creating a procoagulant and prothrombotic environment leading to microvascular thrombi formation, which may lead to multi-organ failure (Liu et al., 2020). Foremost, the clinical results suggest that elevated concentration of circulating D-dimer and other cardiac enzymes, reflecting vascular thrombosis with fibrinolysis, also contribute to pulmonary intravascular coagulopathy as a result of decreased platelet counts (McGonagle et al., 2020). Recent reports hypothesize that the decreased platelet count might be an outcome of the following three mechanisms that is, virus-induced cytokine-mediated destruction of bone marrow resident platelet progenitor cells (low platelet production), dysregulated cytokine-induced platelet destruction, or by microthrombi-induced platelet consumption (Xu et al., 2020). Based upon the viral-mediated inflammatory responses and diagnosis, there are many conventional drug trials ongoing which target the viral structural and nonstructural proteins, in addition to targeting ACE2 and other cytokines (Sanders et al., 2020). It has been earlier reported that the plant metabolites like alkaloids, terpenoids, flavonoids, and lignins (Table 2) have shown their antiviral effects against rotavirus, influenza virus, dengue virus, hepatitis virus, and MERS-CoV as well as SARS-CoV (Swain et al., 2020) (Ghildiyal et al., 2020). These herbal plants through their bioactive compounds can target viral replication and viral entry (Figure 1) (via ACE2 and TMPRSS2), and could be potentially effective against virus-induced complications (Benarba and Pandiella, 2020). Considering all these evidences, the use of traditional herbal plants could be promising either alone or in combination against COVID-19.

**TABLE 1:** List of different key molecules and major complications due to cardiovascular, thrombogenic, and platelet impairments in COVID-19.

	Cardiovascular events	Thrombogenic events	Platelet events
Key molecules	Troponin T (TnT)	vWF	IFN-I
	C-reactive protein (CRP)	TF	Platelet α-granular contents
	Pro-brain natriuretic	ADAMTS-13	Platelet dense-granular contents
	Peptide (BNP)	Fibrinogen	Extracellular vesicle (EV)
	Creatine kinase (CK)	Fibrinogen degradation	
	D-dimer	Products (FDPs)	
	Procalcitonin	D-dimer	
	Ferritin	IL-6	
	IL-6	TNF-α	
	TNF-α		
	IFN-γ		
Major complications	Acute myocardial injury	Venous thromboembolism (VTE)	Thrombocytopenia
	Acute myocarditis	Disseminated intravascular	Platelet hyperactivation
	Myocardial infarction (MI)	Coagulation (DIC)	Platelet–fibrin–microthrombi (PFM)
	Fulminant myocarditis (FM)	Thrombotic	
	Tachy/brady arrhythmia	Microangiopathy (TMA)	
		Intravascular micro/macro	
		Thrombosis (IMT)	
		Blood hypercoagulability	
		Endocardial thrombosis	

**TABLE 2:** List of different phytochemicals having effect on platelet functions and thrombosis, which could have potential roles in the treatment of COVID-19.

Phytochemicals	Species name	Mechanism of action	References
Curcumin	*Curcuma longa* L.	Inhibits ADP, PAF, collagen, and AA-induced platelet activation and aggregation	Kim and Park (2019), Mohd Nor et al. (2016), Soni et al. (2020), Babaei et al. (2020)
Berberine	*Berberis vulgaris* L.	Inhibits platelet by Ca^2+^ mobilization and AA metabolism	Mohd Nor et al. (2016), Kim et al. (2019), Zhang BY et al. (2020)
Magnolol	*Magnolia officinalis* (Rehder and E.H.Wilson)	Inhibits platelet activation and aggregation by inhibiting intracellular Ca^2+^ mobilization and thromboxane formation	Mohd Nor et al. (2016), Lin et al. (2015)
Quercetin	*Allium cepa* L	Inhibits platelet *via* regulation of cAMP/PLA2 pathway and downregulation of intracellular Ca^2+^ mobilization and COX-1	El Haouari and Rosado (2016), Biancatelli et al. (2020)
Andrographolide	*Andrographis paniculata* (Burm.f.) Nees	Inhibits thrombin/collagen-induced platelet aggregation through cGMP/PKG/p38MAPK pathway	El Haouari and Rosado (2016), Enmozhi et al. (2020)
Rutaecarpine	*Tetradium ruticarpum* (A.Juss.) T.G.Hartley	Inhibits of platelet function by inhibiting PLC mediated TXA2 attenuation and downregulation of intracellular Ca^2+^ mobilization	El Haouari and Rosado (2016)
Gingerol	*Zingiber officinale* Roscoe	Inhibits AA-induced platelet activation and aggregation	Mohd Nor et al. (2016), Rathinavel et al. (2020)
Baicalin	*Scutellaria baicalensis* Georgi	Inhibited thrombin production, thrombin catalyzed fibrin polymerization, and platelet functions	Lee et al. (2015), Su et al. (2020)

Here in this review, we are going to overview the effects of SARS-CoV-2 on thrombosis and cardiovascular complications, considering the virology of SARS-CoV-2 and its route of entry. We also highlight the potential therapeutic effects of phytochemicals which possess inhibitory action against platelet function and thrombus formation in order to counter SARS-CoV-2–induced COVID-19.

## Structure and Life Cycle of SARS-CoV-2

**FIGURE 2:**
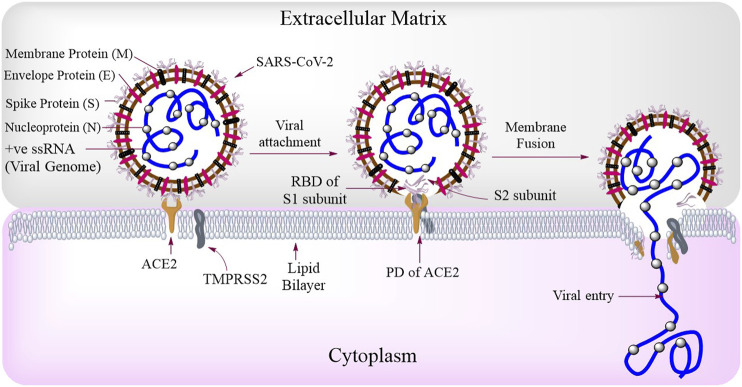
SARS-CoV-2 structure and routes of entry: SARS-CoV-2 consists of Spike protein (S) membrane protein (M), envelope protein (E), nucleoprotein (N), and the viral genome (+ve ssRNA). Among these, the S protein is most important and is made up of two types of subunits that is, S1 (for binding with host receptor) and S2 (for viral fusion) subunits. During viral attachment, the receptor-binding domain (RBD) of S1 interacts with the peptidase domain (PD) of ACE2. The entry and fusion of this virus is aided by a transmembrane serine protease, TMPRSS2.

So far, seven human coronavirus strains have been identified including HCoV-229E, HCoV-OC43, HCoV-NL63, HCoV-HKU1, SARS-CoV, MERS-CoV, and SARS-CoV-2, where the first four causes mild common cold whereas the last three causes severe respiratory syndromes (Xu et al., 2020). Among these last three, SARS-CoV-2–causing COVID-19 is the most lethal one. The genome of SARS-CoV-2 consists of 29,903 bp long positive single-stranded RNA (+ve ssRNA). Each SARS-CoV-2 virion is approximately 50–200 nm in diameter (Ibrahim et al., 2020). Like other coronaviruses, SARS-CoV-2 has four structural proteins that is, S (spike), E (envelope), M (membrane), and N (nucleocapsid) proteins; the N protein holds the RNA genome and the S, E, and M proteins in together forms the viral structure **(** Figure 2 **)**. Apart from these, the genome of this virus also expresses polyproteins, nucleoproteins and membrane proteins that is, RNA polymerase, 3-chymotrypsin-like protease, papain-like protease, helicase, glycoprotein, and accessory proteins (Shereen et al., 2020).

**FIGURE 3:**
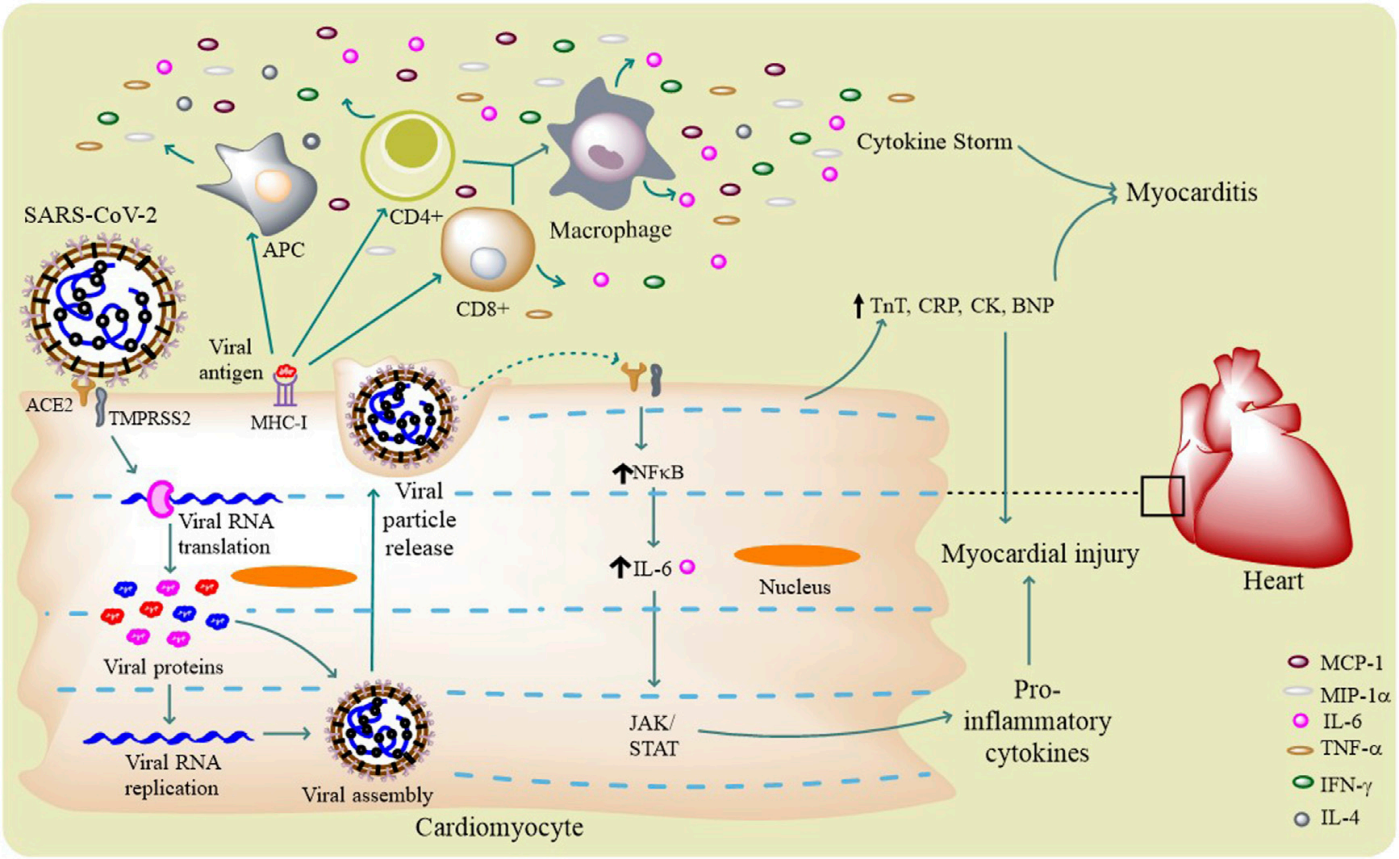
SARS-CoV-2 life cycle and cardiovascular complications: SARS-CoV-2 infects a host cell through ACE2 and TMPRSS2, and through its intracellular life cycle it produces a number of new virions. The virus activates different innate and adaptive immune cells (APCs and T cells) to release certain pro-inflammatory molecules like IL-6, IFN-γ, TNF-α, and MCP-1 that results in cytokine storm. This surge in immunomodulatory molecules mediates the occurrence myocarditis. Virus itself can induce the host cell to produce pro-inflammatory cytokines through IL-6–mediated JAK/STAT and NF-κB pathways. Few circulating biomarker proteins like TnT, CRP, CK, and BNP can potentially cause myocardial injury.

A matured full length spike (S) protein consists of two functional subunits which are responsible for two major functions separately that is, the S_1_ subunit; for binding to the host cell receptor and the S_2_ subunit; for the fusion of viral and cellular membranes (Walls et al., 2020). Between these two subunits, the viral S_1_ protein has receptor-binding domain (RBD) which binds to the peptidase domain (PD) of host cellular receptor ACE2 **(** Figure 2 **)** to facilitate the entry of the virus as seen in case of SARS-CoV (Yan et al., 2020). The spike protein of the virus needs the help of another protein from host cell called transmembrane protease serine 2 (TMPRSS2), for priming of S protein to expose its fusion region to bind with ACE2 (Hoffmann et al., 2020a). The endosomal protease, cathepsin L (CTSL) and Basigin (BSG)/CD147 are also reported to be essential for spike protein processing and viral entry into the host cells (Colaco et al., 2020). After attachment, the viral membrane fuses with the host cell membrane, aided by the S2 subunit of SARS-CoV-2 spike protein. After the fusion of virus and host cell membranes, the viral + ve ssRNA undergoes translation to produce viral replicase polyproteins (PP1a and PP1ab), which are RNA-dependent RNA polymerases (RdRp). The viral RNA polymerases then produce a number of relevant sub-genomic mRNAs, which are further translated into viral proteins **(** Figure 3 **)**. The viral proteins and genomes assemble in ER and Golgi and bud through the ER-Golgi intermediate compartment (ERGIC) to release new virions outside (Shereen et al., 2020).

## The Routes of SARS-CoV-2 Entry: ACE2 and TMPRSS2

As it has been discussed about the structure of SARS-CoV-2 earlier, it is now well elucidated about the basic protein molecules which this virus uses as its receptor(s) to get inside a host cell. In humans, this virus uses two key receptor molecules that is, ACE2 and TMPRSS2 **(** Figure 2 **)** to get the ticket of entry (Hoffmann et al., 2020b). ACE2 is a type I integral membrane glycoprotein of 805 amino acid residues length and functions as a zinc metalloenzyme and carboxypeptidase, found on chromosome X. The genes coded on this chromosome could bring an insight about the gender bias considering the fact that men have only one X chromosome, hence, there is increased fatality for SARS-CoV-2 infection in men as compared to women (Li Y et al., 2020). ACE2 is located abundantly on the surface of the endothelium, kidney, lungs, heart, and intestine (Tikellis et al., 2011). It is a major component of the renin-angiotensin system (RAS), where angiotensinogen is cleaved into a decapeptide, angiotensin I (Ang I) by renin. Then, Ang I can further be cleaved into vasoconstricting octapeptide, angiotensin II (Ang II) by ACE. Then, both Ang I and Ang II can be processed by ACE2 to produce Ang 1–9 and Ang 1–7, respectively. Ang 1–7 peptide, can bind with a G protein-coupled receptor (GPCR) called “Mas” to mediate its vasodilating and vasoprotective effects (Kuba et al., 2013). Here in this way, ACE2 not only antagonizes the effect of Ang II, thus decreasing the blood pressure and inflammation, but also regulates electrolyte balance and cardiovascular homeostasis (Oudit et al., 2003). The *in vivo* studies have demonstrated that ACE, Ang II, and Ang II type 1 (AT1) receptor together functions as lung injury–promoting factors, but the mechanism is still to be deciphered, while the antagonizing regulation of Ang II levels by ACE2 functions as protecting agent against acute and chronic lung injury (Kuba et al., 2013). ACE2 can also cleave a number of other peptides including des-Arg9-bradykinin (DABK), apelin, neurotensin, dynorphin A, and ghrelin (Mughal and O’Rourke, 2018). So in short, an ACE2 comprises an N-terminal peptidase domain (PD) that cleaves Ang I into Ang 1–9, which is further processed into Ang 1–7 and a C-terminal collectrin-like domain (CPD), ending with a 40 amino acid long single transmembrane intracellular segment (Yan et al., 2020).

Similarly, TMPRSS2 is a multidomain type II transmembrane serine protease of 492 amino acid residues length and is found on chromosome 21. TMPRSS2 is expressed predominantly in human prostates, colon, lung, kidney, liver, and pancreas, and it is noticed to be upregulated in prostate cancer. TMPRSS2 can bind and activate protease-activated receptor 2 (PAR2), a GPCR in tumors (Meyer et al., 2013). TMPRSS2 can also cleave and trim the spike proteins of viruses for membrane fusion and entry into different cells especially in the case of SARS-CoV entry into type II pneumocytes aided by ACE2 (Heurich et al., 2014).

## SARS-CoV-2–Induced Cardiovascular Complications

The coronaviruses are not generally restricted to the lungs and associated tissues, but have the potential to invade other organs and tissues, out of which the cardiovascular system, especially the heart and associated blood vessels are most affected. It had been earlier reported that SARS-CoV causes acute myocarditis and other cardiovascular diseases (CVDs) as it takes the help of host ACE2 to get entry (Kuba et al., 2006). Acute myocardial injury, hypotension, tachycardia, bradycardia, cardiomegaly, arrhythmia, and cardiac arrest–like conditions were prevalent in SARS (Xiong et al., 2020). Similarly, MERS-CoV also caused CVDs, where the plasma levels of troponin I and serum creatinine were reported to be elevated (Alhogbani, 2016), (Badawi and Ryoo, 2016). Like SARS-CoV, SARS-CoV-2 also uses this same ACE2 as its receptor for fusion and entry into the host cell. Heart is one of the major sites, where ACE2 is found, thus the virus has a direct impact on the heart also. ACE2 is localized in cardiac endothelial cells, the smooth muscle cells of myocardial vessels, and in cardiac myocytes. This is why there are more number of cardiac complications which are observed in case of COVID-19 (Kuster et al., 2020).

The binding of SARS-CoV-2 with ACE2 can result in the alteration(s) of ACE2 signaling pathways that might lead to acute myocardial injury (Zheng et al., 2020). The myocardial injury is supposed to be manifested by the increase in cardiac troponin I, median creatine kinase (CK) **(** Figure 3 **),** and ACE2-induced cytokine signaling (Kochi et al., 2020). The increased troponin levels are speculated to be possible either by the binding of SARS-CoV-2 to endothelial ACE2–causing coronary microvascular ischemia or due to the virus-induced cytokine storm that induces inflammatory proteins resulting in myocarditis. Studies have reported the high circulatory levels of pro-inflammatory cytokines in patients with severe/critical COVID-19 resulting in acute systemic inflammation of the heart and other organs (Gupta et al., 2020). The presence of viral mRNA in heart and myocardial localizations of coronavirus particles (outside of cardiomyocytes) are evident in a SARS-CoV-2 infected patient suggesting the direct infection the myocardium (Nishiga et al., 2020). The *in vitro* studies have also confirmed this direct infection of cardiomyocytes by SARS-CoV-2 (Bojkova et al., 2020).

Understanding about the mechanism behind cardiac inflammation suggests that cytokine release syndrome (CRS) is evident in COVID-19 condition, but another similar phenomenon also exists in many cancer patients. In cancer many cytokines, more notably, IL-6 was found to be elevated along with ACE2, demonstrating a possible insight of cancer-like surge of cytokines (Gupta et al., 2020). Moreover, the same cytokine (i.e., IL-6) is also seen to be increased in COVID-19 patients (Chen et al., 2020b). Comorbidities are reported to be present, with hypertension being the most common, followed by diabetes and coronary heart diseases. It was recently reported that patients with high plasma troponin T (TnT) levels also possess higher leukocyte counts, lower lymphocyte counts, higher levels of D-dimer, C-reactive protein (CRP), procalcitonin, and N-terminal pro-brain natriuretic peptides (BNPs) (Table 1), leading to myocardial injury (Figure 3) (Guo et al., 2020).

Systemic inflammation and increased shear stress due to increased coronary blood flow can mediate the precipitation of ruptured plaques inside the artery. Platelets generally cover up this ruptured area by clot formation, thus further narrowing the artery diameter, resulting in acute myocardial infarction (MI). Both tachy- and brady-arrhythmias are known to occur in COVID-19 (Bansal, 2020). Due to dysregulated cytokines, there are probabilities for the development of atherosclerotic plaques in SARS-CoV-2 infected patients. The atherosclerotic condition may arise due to elevated concentrations of plasma IL-1β, IL-6, and TNF-α, thus inducing plaque rupture and luminal thrombosis (Vinciguerra et al., 2020). Recent studies have suggested about the infiltration of myocardium by interstitial mononuclear inflammatory cells along with elevated concentrations of Th1 cell response–related cytokines that is, IFN-γ, inducible protein 10 (IP-10), IL-1β, and MCP-1 as well as Th2 cell response–related cytokines that is, IL-10 and IL-4 in COVID-19 autopsy results. These results suggest the possibility of occurrence of SARS-CoV-2–induced fulminant myocarditis (FM) (Chen et al., 2020b).

## SARS-CoV-2–Induced Thrombotic Complications

A report of SARS-CoV-2–induced fibrinous pulmonary thrombi is recently noticed in many patients, along with cardiovascular complications in COVID-19 (Dolhnikoff et al., 2020). Thrombosis refers to the formation of a blood clot inside the blood vessel, which can be an outcome of normal response to injury or due to pathological conditions (i.e., infection or inflammation). Blood platelets along with endothelial cells and coagulation proteins are the pivotal mediators of thrombosis (Koupenova-Zamor et al., 2017)**.** There are basically two types of thrombosis that is, arterial thrombosis that often results in stroke and myocardial infarction (MI), and venous thrombosis which causes venous thromboembolism (VTE) and pulmonary embolism (PE). Arterial thrombi generally form around the ruptured plaques and are rich in platelets, whereas the venous thrombi are rich in fibrin and RBCs (Koupenova-Zamor et al., 2017), (Klatt et al., 2018). Thrombogenic events include ischemic stroke, pulmonary embolism, deep venous thrombosis (DVT), mesenteric ischemia, and acute coronary syndrome, which occur as a result of major complications of certain pathological conditions, such as hypertension, atherosclerosis, and diabetes mellitus (DM) (Fraga-Silva et al., 2009). As we know the SARS virus uses ACE2 as its entry receptor into a cell, ACE2 also plays a major role in the virus-induced thrombogenic events. Contrastingly, it has been reported that thrombus formation is attenuated by the activation of ACE2 and this ACE2 also decreases platelet adhesion to the blood vessels (Fraga-Silva et al., 2009).

Generally, the pro-atherosclerotic Ang II is converted into antiatherosclerotic Ang 1–7 by the help of ACE2 (Wang et al., 2020). Moreover, ACE2 is thought to be responsible for DABK degradation, which is a pro-inflammatory bradykinin that can cause microvascular leakage (Madeddu, 2020). An increase in reactive oxygen species (ROS) and a decrease in endothelium-derived nitric oxide (EDNO) are characteristic features of endothelial dysfunction. Recently, it has been documented that there is a protective RAS signaling axis which is composed of ACE2, Ang 1–7, and Mas receptors, and these molecules together augments the vascular functioning (Fraga-Silva et al., 2013). Many cardiovascular protective molecules through this ACE2-mediated axis are generated that is, NO and Akt phosphorylation, resulting in vasodilation and it also decreases the chance of thrombosis (Rabelo et al., 2011).

In SARS patients, the concentration of D-dimer and prolonged thromboplastin time (PTT) are seen to be elevated according to recent reports. In some other cases, the marantic valvular vegetation (associated with occipital lobe, kidney, spleen, and heart), pulmonary thromboembolism (PTE), and venous thrombosis–like conditions were documented (Ng et al., 2005). Hypofibrinolysis and hypercoagulation were also noticed in SARS patients earlier (Sun et al., 2014). Swelling, proliferation and apoptosis of vascular endothelial cells, inflammatory cell infiltration, edema, and fibrinoid necrosis were also observed in blood vessels of different organs and tissues like muscle, lungs, heart, kidney, adrenal gland, liver, brain, and GI tract of these patients. Moreover, there were evidences for micro circulations of soft tissues around lungs, kidney, and spleen eventually leading to the formation of venous thrombi (Xiang-Hua et al., 2010).

Like SARS patients, the VTE rates are elevated in SARS-CoV-2 infection along with the elevated fibrinogen concentration and high prothrombotic intravascular coagulation (Cavalcanti et al., 2020). The occurrence of cerebral venous thromboses is also reported in COVID-19 patients indicating the possibility of cerebrovascular diseases. The presence of anti–β2-glycoprotein-I IgA and IgG antibodies and anticardiolipin IgA antibodies were also observed in COVID-19 patients. The incidence of elevated prothrombin time and the increased partial thromboplastin time was evident. Reports suggest that thrombocytopenia, leukocytosis, and endothelial injuries were also noticed in these patients (Zhang Y et al., 2020), (Chen et al., 2020a). The concentration of inflammatory cytokines like IL-6 and TNF-α was seen to be elevated causing a prothrombotic condition aided by upregulated tissue factors (TF). But contrastingly, the imbalance in the regulation of antithrombin III, plasminogen activator inhibitor type 1 (PAI-1), and protein C during inflammation and sepsis actually augments an anticoagulated state in case of SARS-CoV-2 infections (Atri et al., 2020). The alteration of fibrinogen and fibrinogen degradation products (FDP) (Table 1) may create the coagulation abnormalities in COVID-19 (Fei et al., 2020).

**FIGURE 4:**
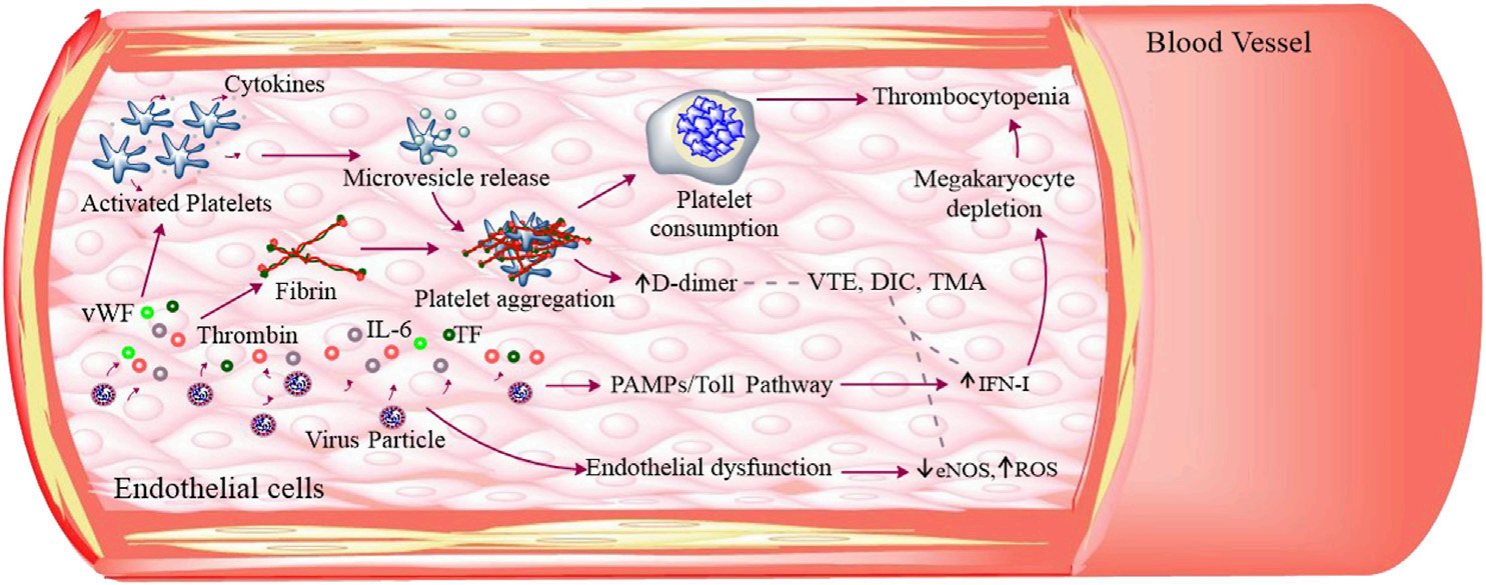
SARS-CoV-2–induced thromboses and platelet dysfunction: SARS-CoV-2 infection caused endothelial dysfunction that releases ROS, IL-6, TF, and vWF. These molecules along with thrombin can activate platelets. The activated platelets adhere, aggregate, and secrete its granular (α and dense granules) contents as well as extracellular vesicles. The aggregated platelets induce high platelet consumption and IFN-I through Toll pathway inhibiting magakaryocyte to produce platelets, thus leading to low platelet production. All these cumulatively results in thrombocytopenia. The fibrinogen degradation produces D-dimer and other thrombogenic molecules that result in venous thromboembolism (VTE), disseminated intravascular coagulation (DIC), and thrombotic microangiopathy (TMA).

These coagulation abnormalities may lead to decreased ADAMTS-13 resulting in aggregation of uncleaved von Willebrand factor (vWF) multimers followed by the deposits of microvascular platelet thrombi, indicating the complications of both disseminated intravascular coagulation (DIC) and/or thrombotic microangiopathy (TMA) (Figure 4) (Levi and Thachil, 2020). The vWF maintains normal hemostasis by mediating platelet–platelet interaction and platelet–blood vessel interaction, but under endothelial injury it can be released into blood circulation as vWF multimers. At the same time, ADAMTS-13, being a metalloprotease in blood circulation can cleave these thrombogenic multimers to ensure an antithrombotic condition (Morici et al., 2020).

In short, the overall coagulopathy observations in COVID-19 patients result in the occurrence of macrovascular thromboses (consisting of RBCs, WBCs, platelets and fibrin) and microvascular thromboses (consisting of platelet and fibrin) in venules, arterioles, and capillaries in all major organs (Becker, 2020). Reports suggest the occurrence of blood hypercoagulability among hospitalized COVID-19 patients (Terpos et al., 2020). The minimal evidences of microangiopathy, endocardial thrombi, viral particles in adipocytes, intravascular megakaryocytes, and an unusual abundance of platelets in the spleen were also observed in SARS-CoV-2 infected patients (Becker, 2020). But, in essence the SARS-CoV-2–induced COVID-19 is a severe prothrombotic disease predominantly (Arachchillage et al., 2020). In addition, many patients who were receiving antithrombotic therapy for thrombotic diseases may have the risk to develop COVID-19 (Bikdeli et al., 2020).

Recent investigations have deduced the role of exosomal non-coding RNAs such as miR-424 and miR-103a, where the former is associated with hypercoagulability and the latter is linked with deep vein thrombosis, although the governing mechanisms is undefined in COVID-19 (Gambardella et al., 2020).

## Role of Platelets in COVID-19

Whenever, the coronavirus-induced thrombosis is noticed, there comes a scenario of virus–platelet interaction. After RBC, platelets are the second most abundant cells found in the blood. Normal platelet counts range from 150,000 to 450,000 platelets/microliter in a healthy person (Izak and Bussel, 2014). There often arises a condition called as thrombocytopenia, in which a significant decrease in the platelet count occurs. This drop in platelet count can occur because of low platelet production, high platelet destruction, and high platelet consumption. Viruses often prefer platelets as their host cells and interact with them for invading the body immunity. The virus attachment occurs *via* different receptors present on platelets like lectins, integrins, and Toll-like receptors (TLRs), causing thrombocytopenia (Assinger, 2014). The release of the alpha and dense-granular contents is often observed from virus-stimulated platelets. These contents are reported to have stimulatory effects on immune cells against viral invasion (Pryzdial et al., 2017).

If coronaviruses are specifically observed, especially SARS-CoV, then thrombocytopenia, lymphopenia, and leukopenia-like conditions are often noticeable. The major reason behind these conditions is the site of viral infection, which are lung tissues and pulmonary endothelial cells. During infection, the damaged cells here cause platelet activation, aggregation and entrapment, resulting in thrombus formation as well as platelet and megakaryocyte consumption (Yang et al., 2004). Many studies suggest that thrombocytopenia, due to SARS-CoV, might have occurred due to platelet consumption (*via* direct viral infection of megakaryocytes and platelets), apoptosis and growth inhibition, natural immune damage of megakaryocytes and platelets, induction of low grade disseminated intravascular coagulopathy (DIC), or due to increased D-dimer (Yang et al., 2005). Like SARS-CoV, MERS-CoV infected persons also show thrombocytopenia (Ko et al., 2016).

There is a strong correlation between the platelet count and mortality of COVID-19 patients (Yang et al., 2020). Later it has been well elucidated from a study including 1799 COVID-19 patients that the viral infection causes a similar decrease in platelet count. It was later proposed that platelet was largely consumed through endothelial damage–mediated platelet activation, aggregation, and thrombosis in addition to abnormal hematopoiesis and alteration of pulmonary capillary bed, considering the fact that lung is the site of platelet release (Lippi et al., 2020). Viral infection often results in overproduction of interferon, which generally possesses antiviral activity. However, type I interferons have the potential ability to inhibit megakaryocytes, resulting in impaired platelet production. More importantly, the lungs may be the sites of platelet release from mature megakaryocytes (Yang et al., 2005). Viral infection, oxygen toxicity, and/or continuing high-pressure, in general, induce lung damage. The damaged lung tissue and pulmonary endothelial cells were supposed to result in platelet activation, aggregation, and entrapment in the lungs, and thrombi formation at the site of injury causing the rapid consumption of platelets and megakaryocytes (Yang et al., 2005).

There have been numerous instances where decreased platelet count (thrombocytopenia) was observed in patients affected by SARS-CoV-2 (Figure 4; Atri et al., 2020; Yin et al., 2020; Hedrich, 2020). Recent case studies have revealed the role of megakaryocyte in this disease pathogenesis, where megakaryocytes were reported to be associated with the formation of platelet-fibrin-microthrombi that were found inside the cardiac microvasculature and other organs (Rapkiewicz et al., 2020). A novel study demonstrated the presence of SARS-CoV-2 specific RNA coding for RdRp, E (Zaid et al., 2020b), and N1 (Manne et al., 2020) in platelets, but the mechanism of entry of these viral factors is still unknown. The above finding leads to the speculation of cellular internalization of viral particle(s) by platelets. In addition to this, an elevated number of phosphatidylserine (PE**)**-exposed extracellular vesicles (EVs) suggesting coagulopahy (Table 1) along with α- and dense-granular contents (Figure 4) of platelets were observed to be released in SARS-CoV-2 infected patients (Zaid et al., 2020b). Moreover, an increased secretion of platelet granular contents that is, PF4 (Platelet Factor 4) from alpha granules and 5-HT (serotonin) from dense granules was observed in COVID-19 patients, indicating the platelet activation. PF4 is considered as a major regulator of T cell development, whereas 5-HT is a weak platelet agonist. If 5-HT is accompanied by other agonists like ADP, it can result in platelet aggregation (Koupenova and Freedman, 2020)**.** The platelets also secreted more soluble CD40L and IL-1β in response to low thrombin treatment, suggesting hyperactivated platelets in SARS-CoV-2 infections in a recent study (Zaid et al., 2020a). But for now, platelets do not have ACE2 receptors for viral binding directly (Battinelli, 2020), hence, a lot about platelet–SARS-CoV-2 interaction has to be elucidated in the near future.

The hyperactivated platelets mediate platelet adherence, aggregation, and secretion (of platelet proteins and few inflammatory cytokines) in COVID-19 suggesting the fact that these platelets are supposed to take part in thrombo-inflammation (Zaid et al., 2020b). This increased platelet activation and aggregation could be manifested by elevated MAPK pathway and generation of thromboxane (TXA) (Manne et al., 2020). Recently, it has been reported that critically affected COVID-19 patients exhibit increased platelet activation and platelet–monocyte aggregation. This platelet–monocyte interaction is responsible for TF expression in the monocytes through P-selectin– and integrin αIIb/β3-dependent signaling (Hottz et al., 2020). There were a number of evidences which suggest that SARS-CoV-2 can directly interact with platelets, thus potentially modulating their thrombotic and inflammatory functions (McFadyen et al., 2020). In future studies, we can hopefully get more clear mechanistic ideas about SARS-CoV-2–induced platelet’s structural and functional abnormalities.

## Role of Immunomodulatory Molecules in COVID-19

Whenever a virus enters a host cell, its antigenic factors are supposed to be presented by antigen presenting cells (APCs) in order to elicit an immune reaction, which is required as body’s natural antiviral approach. Major histocompatibility complexes (MHC-I and II) along with mannose binding lectins (MBLs) are required for antigen presentation which is proven in the case of SARS-CoV (Li X et al., 2020). In general, these viruses adopt some striking approaches for immune evasion. It was well illustrated in case of SARS-CoV and MERS-CoV that these viruses can replicate inside a pattern recognition receptor (PRR)–deprived double membrane vesicle to avoid host defense (Li X et al., 2020).

The coronavirus infections are manifested upon inflammation in different organs and tissues. For these inflammations to take place, cytokines and chemokines play vital roles leading to immunopathology. In COVID-19 patients, the pro-inflammatory cytokines like IL-1, IL-2, IL-6, IL-7, IL-10, IP-10, and TNFα as well as chemokines like IL-8 are found in higher concentrations. The abundance of these immunomodulatory molecules creates cytokine storm, which in turn initiates viral sepsis and inflammation-induced lung and cardiovascular injuries (Qin et al., 2020). By the help of these cytokines and chemokines, the monocytes and T lymphocytes are attracted from the blood to the site of infection and this infiltration of lymphocytes causes lymphopenia and an elevated neutrophil–lymphocyte ratio in COVID-19 patients (Tay et al., 2020).

Apart from these, macrophage inflammatory protein (MIP)-1α, monocyte chemoattractant protein (MCP)-1, C-reactive protein (CRP), G-CSF, procalcitonin, and ferritin were noticed in COVID-19 patients. These are the major inflammatory molecules which can cause myocardial injury (Akhmerov and Marbán, 2020). The presence of C5b-9 (membrane attack complex or MAC), C4d, and mannose binding lectin (MBL)–associated serine protease (MASP-2) were also observed during recent studies, where MAC can mediate microvascular endothelial cell injury and other microthrombotic syndromes (Magro et al., 2020). Hence, the evidences of hyperactivated complement system were observed in recent SARS-CoV-2 infected patients and previous MERS-CoV and SARS-CoV infected ones (Fletcher-Sandersjöö and Bellander, 2020). Recent evidence suggests that acute respiratory distress syndrome (ARDS) is manifested by a number of risk factors, out of which hyaluronan (HA) is important in case of SARS infected patients. The elevated levels of cytokines like TNF-α and Il-1 were noticed in COVID-19 patients. These cytokines have the strong potential to induce the enzyme(s) responsible for HA production and HA-synthase-2 (HAS2) in case of fibroblasts, EpCAM+ alveolar epithelial cells, and CD31+ endothelial cells (Shi et al., 2020). DM is suggested as a potential risk for developing COVID-19 due to elevated concentration of plasma furin (a protease that cleaves S1 and S2 subunits of SARS-CoV-2 spike (S) protein) (Muniyappa and Gubbi, 2020).

In severely affected patients, the activated CD8+ T cells, CD4+ T cells, follicular helper T cells (T_FH_), antibody secreting cells (ASCs), and the antibodies like IgM and IgG were reportedly detected (Thevarajan et al., 2020). But at the same time, the number of helper T cells, suppressor T cells, and regulatory T cells were found to be decreased significantly. SARS-CoV-2 is also associated with increased leukocyte counts, decreased basophil, eosinophil and monocyte counts (Qin et al., 2020). The presence of CCL2 and CCL7 (potent chemokines for the recruitment of monocytes) were noticed to be enriched in bronchoalveolar fluid (BALF) from severe COVID-19 patients (Merad and Martin, 2020). Researchers suggest that the antiviral response is generally induced first by the innate immune cells, by recognizing the pathogen associated molecular patterns (PAMPs) of coronavirus, through TLR-3 and TLR-7, endosomal RNA receptors, cytosolic RNA sensor, and RIG-I/MDA5 (Prompetchara et al., 2020). These recognition events stimulate the downstream NF-κB and IRF3-mediated signaling cascades, inducing the first line of viral defense, the interferon (IFN-I) and other pro-inflammatory cytokine production. Then, the IFN type-I, through JAK-STAT pathway, complexed with IRF9 can induce the expression of IFN-stimulated genes (ISGs) (Prompetchara et al., 2020). It is evident that the SARS-CoV and MERS-CoV interfere with these signaling, leading to the production of IFNs, thus evading the immune system in many ways. As the SARS-CoV-2 uses the same receptor which SARS-CoV uses, the pattern of immunopathology was speculated to be the same. If we take a look at the adaptive immune responses, the T cell response in SARS-CoV suggests that CD8^+^ T cell responses were more of greater magnitude than that of CD4^+^ T cells (Prompetchara et al., 2020). Recent findings also suggest that patients with COVID-19 exhibit elevated levels of neutrophil extracellular traps (NETs) in blood serum (McFadyen et al., 2020). NETs are considered to be very important in platelet aspect as NETs can induce platelet activation *via* TLRs, thus activating the integrin receptor αIIbβ3 that can cause platelet aggregation and granule secretion. Hence, NETs are significant in linking thrombosis, coagulation, and inflammation both in microvascular (local) and macrovascular (systemic) aspects (Ortega-Paz et al., 2020). Many immunological factors and enzymes can be the targets for the development of drugs for COVID-19 (Russell et al., 2020).

## Antiplatelet and Antithrombotic Effects of Phytochemicals in COVID-19

The recent emergence of new variants of SARS-CoV-2 has enforced the use of bioactive components of herbal plants called as phytochemicals. Phytochemicals are the plant derived primary or secondary compounds having health benefits. There are different classes of phytochemicals for example, alkaloids, flavonoids, terpenoids, phenols, and tannins, which possess anti-infection and antioxidant activities (Majnooni et al., 2020)**.** The purified phytochemicals from natural plants were reported to have potential therapeutic effects against coronavirus that is, the herbal extracts from medicinal plants like *Cibotium barometz* (L.) J. Sm.; *Senna tora* (L.) Roxb.; *Dioscorea polystachya* Turcz.; *Gentiana scabra* Bunge; *Taxillus chinensis* (DC.) Danser; and many others were earlier described to inhibit the replication of SARS-CoV genome (Chojnacka et al., 2020). In addition**,** the Indian ayurvedic herb that is, Ashwagandha (*Withania somnifera* (L.) Dunal)-derived Withaferin A (Wi A), a bioactive withanolide, and Withanone (Wi-N) were reported to have inhibitory activities against human papillomavirus (HPV) and influenza viruses which can potentially downregulate the expression of TMPRSS2, thus predicting their action to block SARS-CoV-2 entry (Kumar et al., 2020).

Here in this section, we will discuss only those phytochemicals which have antiplatelet and antithrombotic effects, and can be potentially used for the treatment of COVID-19 (Table 2). As it is already known that cardiovascular complications arise from the impairment of platelet function(s) resulting in platelet hyperactivation and aggregation, causing thrombus formation inside blood vessels (Elisa Hirsch et al., 2017)**.** The following are few phytochemicals which target platelet adhesion, activation, aggregation, and secretion (Figure 1). *Curcuma longa* L. or turmeric is used as an antiviral herb in many areas and is reported to possess anti-inflammatory, antipyretic, and anti-nociceptive properties (Kim and Park, 2019)**.** Curcumin, an active constituent of this plant has various cellular and molecular effects, that is, it inhibits cellular apoptosis, used as an antioxidant, and an ant-tumor and antifibrotic compound. Moreover, this compound has been reported to inhibit ADP, platelet activation factor (PAF), and collagen and arachidonic acid (AA)–induced platelet activation and aggregation (Mohd Nor et al., 2016)**.** Curcumin shows inhibitory effects on TLR, NF-κB, COX, TGF-b1, PAI-1 Cas-3, cytokines, and chemokines. It has been implicated that curcumin has the potential ability to inhibit SARS-CoV and SARS-CoV-2 enzymes, in addition to the evidence of curcumin-mediated inhibition of TMPRSS2 (Soni et al., 2020)**.** Recent reports suggest that curcumin can also be used for the treatment of COVID-19 (Babaei et al., 2020)**.** Similarly*, Berberis vulgaris L.* is also used as an antimicrobial herb and possesses antipyretic as well as antiemetic properties. Berberine is an active constituent of this plant and has been reported to inhibit platelet adhesion and aggregation (Mohd Nor et al., 2016). Berberine inhibits platelet by regulating Ca^2+^ mobilization and arachidonic acid metabolism (Kim et al., 2019). A recent study has revealed that berberine can be effective against SARS-CoV-2, considering the fact that it might be effective in reducing the serum levels of inflammatory molecules to restrict cytokine storm. It has been demonstrated that berberine is effective against influenza by reducing the release oxygen radicals and inhibiting inflammatory injury in mice (Zhang Y et al., 2020)**.** Another plant, *Magnolia officinalis* (Rehder and E.H.Wilson) is used as an anticancer and antipyretic herb. Magnolol is a bioactive constituent of this plant and has proven to inhibit platelet activation and aggregation by inhibiting intracellular Ca^2+^ mobilization and thromboxane formation (Mohd Nor et al., 2016). Magnolol is reported to reduce pro-inflammatory cytokines, ROS production, the expression of iNOS and COX-2, and NF-κB activation in lungs (Lin et al., 2015). Quercetin from *Allium cepa* L. is reported to have antiplatelet activity *via* elevation of cAMP level and attenuation of PLA2 and TXA2 level through downregulation of intracellular Ca^2+^ mobilization and COX-1 (El Haouari and Rosado, 2016). Hence, quercetin in combination with ascorbic acid is speculated to be used for the treatment of COVID-19 (Biancatelli et al., 2020). Andrographolide from *Andrographis paniculata* (Burm.f.) Nees was previously demonstrated to inhibit thrombin-induced platelet aggregation. This compound inhibits platelet aggregation through the upregulation of cGMP/PKG followed by downregulation of p38MAPK/ERK2/NF-κB pathway. Moreover, the compound also attenuated collagen-mediated platelet aggregation through the activation of eNOS/NO/cGMP cascade and eventual inhibition of PI3K/Akt/p38MAPK pathway (El Haouari and Rosado, 2016)**.** A recent research demonstrated that andrographolide can be a potential inhibitor for SARS-CoV-2 main protease in an *in silico* study (Enmozhi et al., 2020). Rutaecarpine from *Tetradium ruticarpum* (A.Juss.) T.G.Hartley was reported to have antiplatelet activity possibly due to inhibition of PLC resulting in TXA2 attenuation and downregulation of intracellular Ca^2+^ mobilization (El Haouari and Rosado, 2016). In addition, the *Ginkgo biloba* L. extract inhibited platelet function by inhibiting the production of TXA2, and activated the production of cAMP which ultimately reduced platelet intracellular Ca^2+^ levels (El Haouari and Rosado, 2016). The extract of *Carthamus tinctorius* L. is reported to have antithrombotic activity as it increases activated partial thromboplastin time (APTT), plasma thrombin time (PTT), and prothrombin time (PT). A phytochemical named gingerol from *Zingiber officinale* Roscoe has also been reported to have antiplatelet activity as it inhibits AA-induced platelet activation and aggregation probably through inhibition of COX-1 (Mohd Nor et al., 2016). It has been recently reported that 6-gingerol has good pharmacokinetic properties and could be a promising phytochemical against SARS-CoV-2 through *in silico* studies (Rathinavel et al., 2020). Baicalin from *Scutellaria baicalensis* Georgi has been demonstrated to inhibit thrombin catalyzed fibrin polymerization and platelet functions. It also inhibited the FXa and thrombin productions, and also decreased TNF-α–induced PAI-1 production (Lee et al., 2015). Baicalin is recently reported to be a novel inhibitor of 3CL protease of SARS-CoV-2 (Su et al., 2020). There are some bioactive phytochemicals like Magnolol and Rutaecarpine whose antiplatelet effects are well elucidated (El Haouari and Rosado, 2016), (Mohd Nor et al., 2016), but their therapeutic potential against SARS-CoV-2 is still to be experimentally validated. Besides, there are other phytochemicals which are not illustrated here having antiplatelet and antithrombotic activities and their potential efficacy is still to be proven against COVID-19. Thus, *in vitro* and *in vivo* studies are still needed for the evaluation of pharmacokinetic properties and deleterious potential of these phytochemicals in COVID-19.

## Conclusion

The SARS-CoV-2 is not always restricted only to the lungs and associated tissues, but it can invade other organs and tissues, out of which the heart is affected most. Severe respiratory distress and lung associated complications were initially thought to be the main cause of COVID-19 related deaths. Later on, detailed research and the discovery of many biomarkers of cardiac injury and its related complications were also found to be associated with the high fatality rate in COVID-19 patients. The detection of anti–β-2 glycoprotein IgA and IgG antibodies along with anticardiolipin IgA in SARS-CoV-2 infected patients shows inappropriate blood clot formation leading to cardiovascular complication. Thrombocytopenia, endothelial dysfunction, thromboembolism (Table 1) and leukocytosis were some of the underlying causes of the severe acute illness in COVID-19 patients. Platelet is an essential innate immune cell which responds to the SARS-CoV-2 infection. Viral infection–induced lung tissue damage can result in platelet activation, aggregation, entrapment in the lungs, and thrombi formation at the site of injury. Reduced platelet count was associated with their direct consumption during viral infection, increased number of D-dimers from fibrinolysis, high levels of type I interferon, and DIC.

The virus-induced cytokine storm causes severe inflammation in the lung tissues and increases the risk of cardiovascular tissue damage. Platelets were also reported as carriers for viral antigens that can respond to infection by secreting cytokines, granular contents, and EVs. Briefly, thrombocytopenia or low platelet count is associated with abnormal coagulation function(s), severe disease manifestation, and increased mortality in patients with COVID-19. In future, experiments in this aspect can bring insights for other roles of platelet in SARS-CoV-2 infection too. Hence, specific consideration ought to be given to cardiovascular insurance during the treatment for COVID-19. There are a number of conventional drug and vaccine trials going on for the treatment of COVID-19, but the natural bioactive compounds were earlier proven to be effective against SARS-family viruses. Phytochemicals are the natural bioactive components of herbal plants that were reported to have various cardioprotective activities, including the inhibition of platelet adhesion, activation, aggregation, and secretion, thus reducing the possibility of thrombus formation and vascular occlusion (Figure 1). These phytochemicals in general can target the platelet intracellular Ca^2+^ mobilization, thromboxane synthesis, and phospholipases–mediated MAPK pathways resulting in the downregulation of platelet functions. Hence, such phytochemicals could be advantageous and effective in the treatment of COVID-19. Further *in vitro* and *in vivo* studies on the efficacy of these phytochemicals will validate their effectiveness in COVID-19–induced thrombotic and cardiovascular complications.
